# Improving the Mapping of Smith-Waterman Sequence Database Searches onto CUDA-Enabled GPUs

**DOI:** 10.1155/2015/185179

**Published:** 2015-08-03

**Authors:** Liang-Tsung Huang, Chao-Chin Wu, Lien-Fu Lai, Yun-Ju Li

**Affiliations:** ^1^Department of Medical Informatics, Tzu Chi University, Hualien 970, Taiwan; ^2^Department of Computer Science and Information Engineering, National Changhua University of Education, Changhua 500, Taiwan

## Abstract

Sequence alignment lies at heart of the bioinformatics. The Smith-Waterman algorithm is one of the key sequence search algorithms and has gained popularity due to improved implementations and rapidly increasing compute power. Recently, the Smith-Waterman algorithm has been successfully mapped onto the emerging general-purpose graphics processing units (GPUs). In this paper, we focused on how to improve the mapping, especially for short query sequences, by better usage of shared memory. We performed and evaluated the proposed method on two different platforms (Tesla C1060 and Tesla K20) and compared it with two classic methods in CUDASW++. Further, the performance on different numbers of threads and blocks has been analyzed. The results showed that the proposed method significantly improves Smith-Waterman algorithm on CUDA-enabled GPUs in proper allocation of block and thread numbers.

## 1. Introduction

Sequence alignment is one of the most important methodologies in the field of computational biology [1]. It describes the way of arrangement of DNA/RNA or protein sequences, in order to identify the regions of similarity among them and to infer structural, functional, and evolutionary relationship between the sequences. Sequence alignment enables researchers to compare the sequences of genes or proteins with unknown functions to sequences of well-studied genes or proteins. When a new sequence is found, the structure and function can be easily predicted by performing sequence alignment because a sequence sharing common ancestor would exhibit similar structure or function.

The most widely used sequence alignment algorithm may be the Smith-Waterman algorithm that was first proposed by Smith and Waterman in 1981 [2] and optimized by Gotoh in 1982 [3]. It performs local sequence alignment, which is designed especially for dissimilar sequences that are suspected to contain regions of similarity or similar sequence motifs within their larger sequence context. To determine similar regions between two nucleotide or protein sequences, the Smith-Waterman algorithm, instead of looking at the total sequence, compares segments of all possible lengths and optimizes the similarity measure. The Smith-Waterman finds the alignment in a more quantitative way by giving scores for matches and mismatches for every possible pair of residues. The scores are predefined in scoring matrices, such as PAM (point accepted mutation) [4] and BLOSUM (blocks substitution matrix) [5]. In general, a positive score is assigned for a match, a negative score for a mismatch, and a negative score for a gap penalty.

Although the Smith-Waterman algorithm is one of the most advanced and sensitive pairwise sequence comparison algorithms currently available, it is theoretically about 50 times slower than other popular heuristic-based algorithms, such as FASTA [6, 7] and BLAST (Basic Local Alignment Search Tool) [8, 9]. The Smith-Waterman algorithm is slow because it imposes no constraints on the alignment; that is, no sequences will be filtered out if the final alignment score is not above a predefined threshold. However, the Smith-Waterman algorithm is still widely used because of its high sensitivity of sequence alignment even though it has higher time complexity of algorithm. To enable the Smith-Waterman algorithm to produce exact results in a reasonably shorter time, much research has been focusing on using various high-performance architectures to accelerate the processing speed of the algorithm [10–27]. In particular, it becomes a recent trend to use the emerging accelerators and many-core architectures, such as field-programmable gate arrays (FPGAs) [10–12], cell/BEs [13–17], and general-purpose graphics processing units (GPUs), to run the Smith-Waterman algorithm [18–26].

FPGAs allow customers to configure large resources of logic gates and RAM blocks to implement complex digital computations after manufacturing. Since an FPGA can be configured to execute the Smith-Waterman algorithm, it can be regarded as special-purpose hardware for the Smith-Waterman, resulting in high execution speed. Note the FPGA-based implementation of the Smith-Waterman is more hardware centric. Cell/BEs are multicore microarchitecture that combines a general-purpose power architecture core with streamlined coprocessing elements. The primary feature of cell/BEs is to greatly accelerate multimedia and vector processing applications by introducing the streaming SIMD extensions 2 (SSE2) technology. SIMD instructions can greatly increase performance when exactly the same operations are to be performed on multiple data objects. The SIMD instructions on cell/BEs are used by several research projects to parallelize the Smith-Waterman algorithm.

Modern general-purpose GPUs are not only powerful graphics engines but also highly parallel programmable processors. Today's GPUs use hundreds of parallel processor cores executing tens of thousands of parallel threads to rapidly solve large problems, now available in many PCs, laptops, workstations, and supercomputers. Because of the availability and the popularity, GPUs have been used to implement the Smith-Waterman algorithm, where CUDASW++ is the leading research that provides the fast, publicly available solution to the exact Smith-Waterman algorithm on commodity hardware [18–20]. CUDASW++ 3.0 is the latest version, which couples CPU and GPU SIMD instructions and carries out concurrent CPU and GPU computations [20].

This study aimed at how to improve CUDASW++, especially for short query sequences. Since we observed that the shared memory in each streaming multiprocessor is not fully utilized in CUDASW++, the execution flow of the Smith-Waterman algorithm was rearranged to fully utilize the shared memory for reducing the amount of slow global memory access. This paper is organized as follows.  Section 2 introduces CUDASW++ and CUDA-Enabled GPUs.  Section 3 presents our method to map the Smith-Waterman database search algorithm onto a CUDA-Enabled GPU for short query sequences.  Section 4 demonstrates the experimental results and analyse the performance. Finally, conclusions are given in Section 5.

## 2. Related Work

CUDASW++ is one of the key projects for implementing the Smith-Waterman sequence database search algorithm on general-purpose GPUs, where the source code of CUDASW++ is publicly available [18–20]. Liu et al. have proposed three versions of CUDASW++ so far, to map the Smith-Waterman database search algorithm onto nVIDIA GPUs. CUDASW++ 1.0 completes all the Smith-Waterman computations on GPUs by fully exploiting the aggregate power of multiple G200 (and higher) GPUs [18].

CUDASW++ 2.0 aims at optimizing the performance of CUDASW++ 1.0 based on the SIMT abstraction of CUDA-enabled GPUs [19]. Two optimization approaches have been implemented in CUDASW++ 2.0. In the first approach, the authors defined a length threshold to partition the database into two parts. For those sequences of length shorter than the threshold, CUDASW++ 2.0 adopts the intertask parallelization method for their alignments with the query sequence. For the other sequences, the system uses the intratask parallelization method. The intertask parallelization method uses one thread to align one subject sequence with the query sequence. It means that multiple subject sequences are aligned with the query sequence concurrently, without interthread communication. On the other hand, the intratask parallelization method uses all the threads in a block to align one subject sequence with the query sequence. Since the intratask parallelization method imposed communication and synchronization among threads, it has better performance than the intertask method only for the sequences of lengths larger than the predefined threshold. According to the statistics, over 99% of the subject sequences will be aligned by the interthread parallelization method. Our work focuses on improving the inter-task parallelization method due to this observation.

The second approach proposed in CUDASW++ 2.0 is the column-major parallelization method. Similar to the intratask method, all the threads in a block work together to align one subject sequence with the query sequence. However, the column-major method aims to exploit more thread parallelism by speculative computation. That is, threads start the computation of *H* scores before the dependent data, *F* scores, are available. The speculative computation assumes the speculative *H* scores will be larger than or equal to *F* scores. Since it is speculative computation, the lazy-*F* loop is used to verify whether the assumption is correct for each *H* score or not. If any answer is false, all the *H* scores on the same column have to be recalculated. According to the evaluation results reported, the column-major method outperforms the first approach only for few cases. In practice, CUDASW++ adopts the first approach to perform the alignment.

CUDASW++ 3.0 is written in CUDA C++ and PTX assembly languages, targeting GPUs based on the Kepler architecture. It conducts concurrent CPU and GPU computations to accelerate the Smith-Waterman algorithm [20]. According to the compute powers of the CPU and the GPU used in the system, CUDASW++ 3.0 dynamically distributes all sequence alignment workloads over CPUs and GPUs to balance the runtimes of CPU and GPU computations. On the CPU side, the streaming SIMD extensions- (SSE-) based vector execution units and multithreading are employed to speed up the Smith-Waterman algorithm. On the GPU side, PTX SIMD video instructions are used to parallelize the Smith-Waterman algorithm.

Manavski and Valle introduced the idea of query profile and the sorted database [21]. Ligowski and Rudnicki reported their research result almost at the same time as CUDASW++ 1.0 and they investigated how to use shared memory to improve the performance of the Smith-Waterman algorithm [22]. The above two projects both did not exploit the intratask parallelism for long subject sequences. Khajeh-Saeed et al. proposed an interesting parallel scan Smith-Waterman algorithm [23]. They argued that the classic diagonal parallelization approach suffers from nonuniform parallelism distribution across phases of dynamic programming and the memory access pattern is hard to the advantage of memory coalescing. Instead, they aimed to fully parallelize the computation of the cells in one row of the dynamic programming matrix at the same time. To enforce the data dependence between the cells in the same row, they needed to perform the parallel scan to update the values of the cells, resulting in high synchronization overhead between threads and blocks. The parallel scan algorithm can be used in the intratask kernel of CUDASW++. Blazewicz et al. mainly focused on how to improve the backtracking procedure of the Smith-Waterman algorithm [24]. They proposed to use four Boolean matrices to indicate the proper direction of backward moves for every position during the process of backtracking. Their method can be adopted by other packages for further performance improvement, including CUDASW++. Hains et al. proposed using a tiling approach to improve the performance of the intratask kernel of CUDASW++ [25]. They also pointed out several important design issues for tuning performance, including how to ensure that registers are used, instead of global memory, even when the capacity is not exceeded.

**Figure 1:**
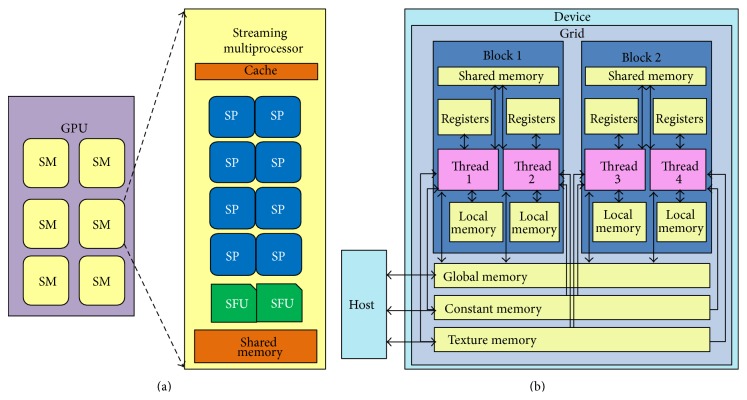
The block diagram of (a) CUDA-enabled GPUs and (b) the memory hierarchy.

CUDA is a new language and development environment, allowing execution of general-purpose applications on NVIDIA's GPUs [28]. The hardware model is comprised of several highly threaded streaming multiprocessors (SMs), where each SM consists of a set of streaming processors (SPs), as shown in Figure 1(a). The computing system consists of a host that is a traditional CPU, also called* host*, and one or more GPUs, also called* device*, as shown in Figure 1(b).

## 3. Materials and Methodology

### 3.1. Multiple Subject Sequences of Parallel Method on Smith-Waterman Algorithm

The Smith-Waterman algorithm has been mathematically proven to find the best local alignment of two sequences. The algorithm compares two sequences by computing a score that represents the minimal cost of transforming one sequence to another using two elementary operations: match/mutation and insertion/deletion. If two characters from two sequences match, the cumulative score is increased. However, if one character in the first sequence can be mutated from another character in the second sequence, the cumulative score is either increased or decreased depending on the relationship between these two characters defined in the adopted substitution matrix. There are different substitution matrices for scoring alignment of two sequences. For instance, a BLOSUM (blocks substitution matrix) is a substitution matrix used for sequence alignment of proteins and it records a score for each of the 210 possible substitution pairs of the 20 standard amino acids. Several sets of BLOSUMs exist using different alignment database, where each is named with a number. For two sequences, *S*_1_ and *S*_2_ with lengths *L*_1_ and *L*_2_, the first elementary operation, match/mutation, computes the similarity *H*(*i*, *j*) of these sequences ending at positions *i* and *j* in order to identify common subsequences. We call the sequence *S*_1_ the query sequence and the sequence *S*_2_ the subject sequence. The computation of *H*(*i*, *j*), for 1 ≤ *i* ≤ *L*_1_ and 1 ≤ *j* ≤ *L*_2_, is formulated by the following recurrences:

(1) Deletion  Ei,j=max⁡Ei,j−1,Hi,j−1−ρ−σ,Insertion  Fi,j=max⁡Fi−1,j,Hi−1,j−ρ−σ,Similarity  Hi,j=max⁡0,Ei,j,Fi,j,Hi−1,j−1+sbtS1i,S2j,

where sbt(*S*_1_[*i*], *S*_2_[*j*]) represents the score for the *i*th character in the sequence *S*_1_ and the *j*th character in the sequence *S*_2_ defined in the specified substitution matrix. If *S*_1_[*i*] and *S*_2_[*j*] are the same character, they are matched; otherwise, it is assumed that the two are derived from an ancestral character, that is, mutation.

Furthermore, in the recurrences, *E*(*i*, *j*) and *F*(*i*, *j*) represent the two cases when gaps are inserted because of different sequence length, where a gap is a consecutive run of spaces in an alignment, represented as a dash on a protein/DNA sequence alignment. To perform a sequence alignment, we write one sequence on top of another, where the characters in one position are deemed to have a common evolutionary origin. If the two sequences are of different lengths, gaps are inserted to make them of equal length. Gaps are used to create alignments that are better conformed to underlying biological models and more closely fit patterns that one expects to find in meaningful alignments. A gap can be inserted into either a query sequence or a subject sequence. Since an insertion in one sequence can always be seen as a deletion in the other one, when a gap is used in the query sequence, it is a gap insertion; otherwise, it is a gap deletion. Gap penalty values are designed to reduce the score when a sequence alignment has been disturbed by gaps. An initial penalty is assigned for a gap opening, *ρ*, and an additional penalty is assigned for gap extensions that increase the gap length, *σ*.

We investigate the problem of aligning each subsequence in a database with the query sequence using the Smith-Waterman algorithm, where the database consists of *N* subject sequences. The problem can be divided into *N* independent subproblems, where each subproblem is to use the Smith-Waterman algorithm to align the query sequence and one subject sequence. Basically, we solve the problem in a way that is the same as CUDASW++ 2.0. That is, a thread will be assigned to align one subject sequence with the query sequence if the length of the subject sequence is not larger than the user predefined threshold. However, how each thread uses the memory resources on a CUDA-enabled GPU in our method is different from that in CUDASW++ 2.0. On the other hand, for those subject sequences of lengths larger than the threshold, all the threads in a block will perform the alignment in parallel for only one subject sequence. For simplicity, the details of how to use multiple threads to perform an instance of the Smith-Waterman algorithm are omitted.

### 3.2. Thread Assignment and Sequence Alignment in CUDA-Enabled GPU

We assign one thread for solving one subproblem in a CUDA-enabled GPU. The advantage of such a thread assignment is no interthread communication incurred. Because of the different lengths of the subject sequences in the database, the execution times of the threads are not the same. Therefore, the execution times of threads in a warp will be different and the warp cannot be complete until the slowest thread finishes its work, resulting in a longer warp execution time. Since a block will execute warps one by one, longer warp execution times lead to longer block execution time. To address the problem, the subject sequences allocated to the same warp should be of similar length. To meet this requirement, we preprocess the database by sorting the subject sequences by the length in the ascending order. At the run time, every 32 continuous subject sequences will be assigned to one warp.

Even though the sorted subject sequences can shorten the execution times of warps, the sorted subject sequences cannot be accessed efficiently in the global memory. The reason is explained as follows. Because CUDA-enabled GPUs are SIMD architecture, the 32 threads in a warp will access the *i*th characters from their assigned subject sequences in parallel, respectively. However, subject sequences are stored one by one in the global memory, resulting in that every 32 *i*th elements in 32 continuous subject sequences are not stored contiguously. Therefore, the 32 threads in the same warp cannot access the 32 *i*th elements in one bus transaction. To conquer this problem and to take the advantage of memory coalescing for accessing global memory, the sorted subject sequence database is transformed based on the following method before sending subject sequences to the global memory on a GPU. The elements from each 32 continuous subject sequences are stored interleavingly. In other words, the *i*th elements from the *k*th sequences in every 32 continuous subject sequences are stored at (32 × *i* + *k*)th memory location.

To align the query sequence with each subject sequence in the database, the query sequence will be used repeatedly. On the other hand, the number of amino acids is only 20. That is, each character of the query sequence will be pairing with the 20 amino acids repeatedly. It is time consuming if each time of pairing has to access the substitution matrix one tome for scoring. Therefore, it is usual to construct a query profile to address the problem. A query profile is a two-dimensional array. The row fields consist of the characters of the query sequence in order and the column fields consist of the 20 amino acids. The value of each cell of a query profile is the score of the relationship between the corresponding amino acid and the corresponding character in the query sequence. The query profile is saved on the texture memory. Each time we can fetch 4 consecutive scores from the texture memory in one access and use four registers to save the 4 scores in a vector fashion. In this way, the cost of accessing the scores can be reduced.

### 3.3. Memory Allocation at Run Time

**Figure 2:**
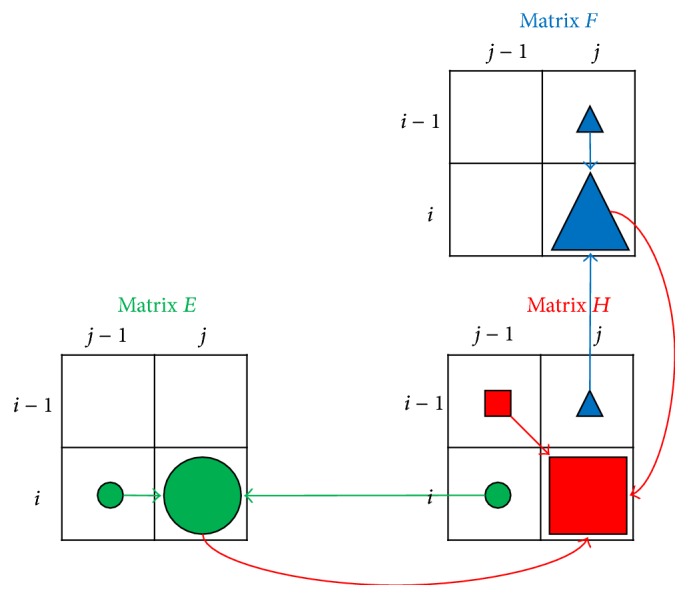
The dependence relationship between matrices *H*, *E*, and *F*.

To perform the Smith-Waterman algorithm, at run time we need to allocate memory space for the three matrices: *H*, *E*, and *F*. Since the matrices might be very large but the data are intermediate, it is better not to save all the matrices data in the global memory. To reduce the required memory space as much as possible, we analyze the dependency relationship among the three matrices, as shown in Figure 2. To compute the element, *H*(*i*, *j*), we require to access the values of *E*(*i*, *j*) and *F*(*i*, *j*), meaning that we have to calculate *E*(*i*, *j*) and *F*(*i*, *j*) before *H*(*i*, *j*) in the same loop iteration. On the other hand, *E*(*i*, *j*) depends on *E*(*i*, *j* − 1) and *H*(*i*, *j* − 1) while *F*(*i*, *j*) depends on *F*(*i* − 1, *j*) and *H*(*i* − 1, *j*). It means that, when executing iteration (*i*, *j*), we require the intermediate data produced in iterations (*i* − 1, *j*) and (*i*, *j* − 1) only. Therefore, we have no need to save all the intermediate data of matrices *H*, *E*, and *F* on global memory. Instead, we can use registers to save the intermediate data produced in the previous iterations and the computation result in the current iteration. Assume the index of the inner loop is *j*; two registers are sufficient for storing *E*: one for *E*(*i*, *j* − 1) and one for *E*(*i*, *j*). However, a row of registers is required for *F*, which is infeasible because of very limited number of registers for a thread. Similarly, we need a row of registers plus two for *H*. Due to the limited number of registers available in a thread, we use shared memory to buffer the spilled values of registers.

However, all the threads in a block, on a streaming multiprocessor, share the shared memory and its space is not as large as the global memory's. For instance, the amount of shared memory on C1060 is 16 K bytes. If there are 256 threads in a block, each thread can have 64 bytes, that is, 16 words. Therefore, we can swap at most 16 register values out to shared memory for each of the threads. Otherwise, the shared memory is overflow and the slow global memory has to be used to buffer the data due to overflow, which will degrade the overall performance. Therefore, we use our method only when shared memory can store all the spilled register values. In other cases, the original CUDASW++ will be invoked to process the query. Our method will be built in the CUDASW++ package as an execution option. In general, if there are *T* threads in each block and the amount of available shared memory is *S* bytes per streaming multiprocessor, the longest length of one query sequence is equal to *S*/(4 × *T*) since matrices *H* and *E* require shared memory for buffering and each cell requires two bytes.

### 3.4. Proposed Mapping Algorithm on CUDA-Enabled GPUs

**Figure 3:**
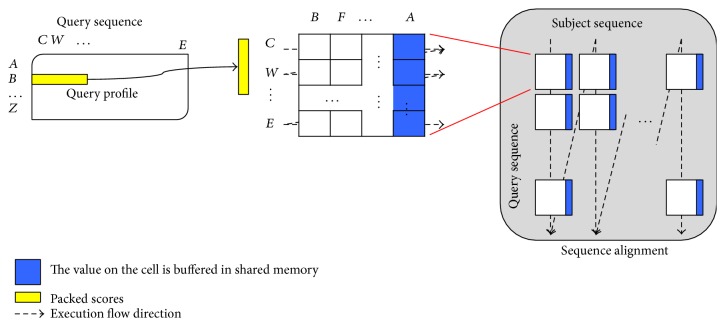
The overview of the proposed mapping algorithm.

Registers are the fastest memory and shared memory is faster than global memory one hundred times. To use registers to solve dependence as much as possible and to efficiently use shared memory to buffer spilled registers, we propose the following algorithm to perform the Smith-Waterman algorithm, as shown in Figure 3. Every *K* consecutive residue on the assigned subject sequence for a thread is grouped in order, padding dummy residues at the end of the subject sequence when necessary. Similarly, every *P* consecutive residue on the query sequence is in an ordered partition, padding dummy residues at the end of the query sequence when necessary. A tile is defined as the alignment of one group of ordered residues on the subject sequence with a partition of ordered residues on the query sequence. To enforce the dependence, tiles will be aligned one by one in the column-major order, where the tiles on the same column are processed from top to bottom. Furthermore, for a tile, the first residue on the query sequence is aligned with the *K* residues on the subject sequence one by one, from left to right; then the second, the third, and to the *P*th residues are aligned with the *K* residues on the subject sequence, respectively.

For each tile, the values on the *K*th column have to be read by the tile next to it on the right hand side. Similarly, the values on the *P*th row have to be read by the tile next to it to the bottom. For each of the *F* and *H* matrices, we use *K* registers to save *K* consecutive values on the same row, respectively. To calculate the next row, the *K* registers can be reused because the source operand and the destination operand of an instruction can use the same register. Therefore, the values on the *P*th row of a tile can be forwarded through registers to the first row on the next tile, right below the tile. However, the values on the *K*th column cannot be forwarded through registers to the next tile on the right hand side because the *K*th register is reused to save the value for the next row on the *K*th column, even in the same tile. Note that the next tile on the right hand side of the current tile is not the next tile to be processed; it is the next tile on the bottom of the current tile. Consequently, shared memory is used to buffer *P* values on the *K*th column for each tile. The buffered values can be read to calculate the values for the first column of the tile on a tile's immediate right hand side.

Since tiles are processed in column-major order, the shared memory for buffering the *K*th column of tiles on the same column can be reused when the next column of tiles is processed. If the query sequence length is not too long, shared memory can buffer all the values on the *K*th column of tiles on the same column, no global memory access is required. Let the number of bytes per shared memory be *X*, the number of threads per block be *T*, and the number of residues in the query sequence be *Q*. If *X* ≥ *T* × *Q*, no global memory access is required. At the run time, the system can decide if shared memory can buffer all required values on a column based on the hardware configuration and the length of the query sequence specified by users. Since the shared memory is rather limited, the query sequence length cannot be too long for applying our method.

Since the query profile packs the scores of every four continuous residues and saves the packed scores on texture memory, we can get four scores whenever texture memory is accessed. The fetched four scores have to be saved in four registers because these scores are for the calculation of the four consecutive cells on the same column inside a tile. Consequently, *P* should be a multiple of four. The pseudo code of our method is shown in Pseudocode 1, where *P* and *K* are set to four to reduce the pressure on the register requirement.

## 4. Results and Discussion

### 4.1. Analysis of Experimental Platforms between Tesla C1060 and Tesla K20

We used CUDA version 5.0 to extend CUDASW++ 2.0 with our proposed method. Two platforms are used to evaluate our proposed method. The Dell Precision T5500 computer workstation is our first experimental platform, consisting of one Intel Xeon CPU and one nVIDIA Tesla C1060 GPU. Another experimental platform is comprised of Intel Core i7 CPU and nVIDIA Tesla K20, where Tesla K20 is based on the new Kepler architecture, providing a 15-time increase in double precision performance compared Tesla C1060. Tesla K20 consists of 13 streaming multiprocessors with 2496 cores totally while Tesla C1060 has 30 streaming multiprocessor with 240 cores totally.

The Kepler architecture employs a new streaming multiprocessor architecture, called SMX, which deliver more processing performance and efficiency. An SMX allows a greater percentage of space to be applied to processing cores versus control logic. In addition, the Kepler architecture simplifies GPU programming by allowing programmers to easily accelerate all parallel nested loops, resulting in a GPU dynamically spawning new threads on its own without going back to the CPU. Finally, the Kepler architecture also uses the Hyper-Q technique to slash CPU idle time by allowing multiple CPU cores to simultaneously utilize a single Kepler GPU.

The hardware configurations of Tesla C1060 and K20 are shown in Tables 1 and 2, respectively. The operating system installed is Linux and its version is Ubuntu 12.04 64-bit. The BLOSUM64 protein sequences database is used for the performance evaluation. Moreover, the short query sequences are also from the BLOSUM 64.

### 4.2. Performance Evaluation on Tesla C1060

**Figure 4:**
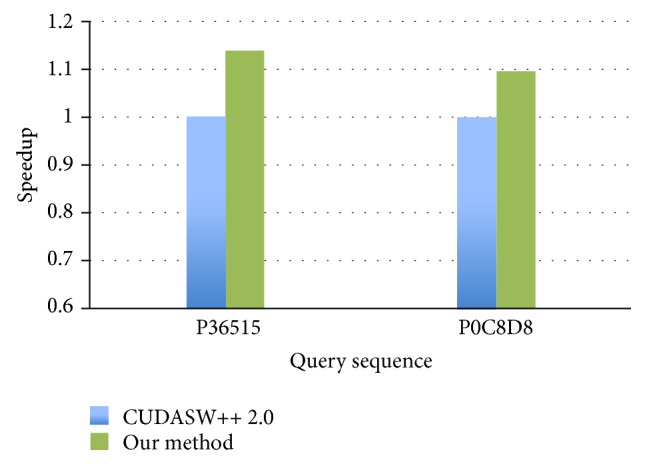
The speedup of our method over CUDASW++ 2.0 on Tesla C1060 with 256 blocks.

We present the speedup of our method in the following, where the speedup is to divide the execution time of a method by the execution time of CUDASW++ 2.0. The performance improvement of our method over CUDASW++ 2.0 is shown in Figure 4. The C1060 has 16 K-byte shared memory. Because there are 256 threads in each block, each thread is assigned with 64-byte, that is, 16-word, shared memory for storing the matrices *H*, *E*, and *F*. The best speedup is about 1.14 when the query sequence is P36515 consisting of four amino acids.

### 4.3. Performance Evaluation with CUDASW++ 2.0 and CUDASW++ 3.0 on K20

**Figure 5:**
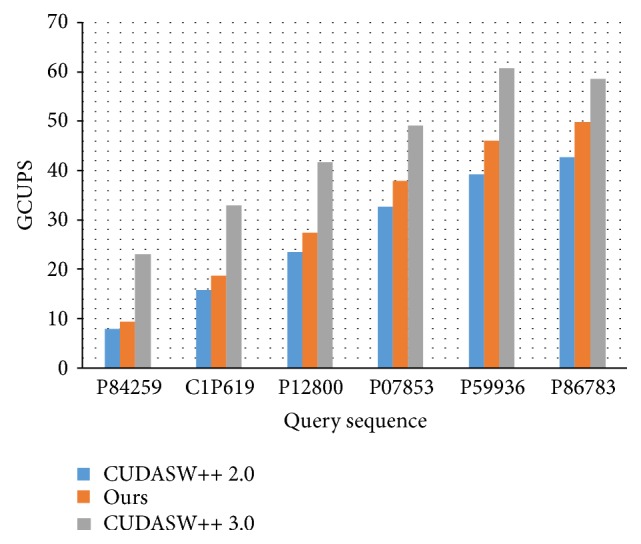
The GCUPS comparison of our method with CUDASW++ 2.0 and CUDASW++ 3.0 on Tesla K20.

We further compared with different methods on Tesla K20 with 64 blocks and 64 threads. On the Kepler GPU, K20, the space of shared memory per streaming multiprocessor is much larger than that on Tesla C1060. This characteristic can store more spilled register values for CUDASW++ series and thus reduce the frequency of swapping some 10 shared memory values out to/in from the slow global memory. The feature enables our method to process longer query sequences with more parallel threads per block. The GCUPS comparison between our method and CUDASW++ 2.0 as well as CUDASW++ 3.0 is shown in Figure 5, where GCUPS stands for giga cell updates per second. Our method outperforms CUDASW++ 2.0 for all of the query sequences because ours can fully utilize the shared memory without the need of swapping data between shared memory and global memory. When the query sequence length becomes larger, our method can provide more performance improvement. The reason is because CUDASW++ 2.0 required more data swapping between shared memory and global memory when processing a longer query sequence.

However, our method could not outperform CUDASW++ 3.0 because the latter performed the sequence alignment by coupling both the compute powers of CPU and GPU while our method was built upon CUDASW++ 2.0, which utilized the GPU compute power only. That is, the subject sequences were divided and allocated to CPU and GPU according to each individual compute power. On the GPU side, CUDASW++ 3.0 used PTX assembly instructions to implement the key recurrence equation and procedure of finding the optimal local alignment score, where every assembly instruction operated on quads of 8-bit signed values, corresponding four independent alignments. The idea of our proposed method can be applied to CUDASW++ 3.0 in the future to accelerate the processing of alignments with short query sequences for the intertask kernel.

CUDASW++ 3.0 used local memory to buffer one row of matrices *H* and *E* while we used shared memory instead for the buffering because the access latency of local memory is much longer than that of shared memory. However, the shared memory space is so much smaller than the local memory space that it is impossible to only use shared memory for all the buffering without the help of global memory or local memory if sequences are too long. That is why our method is applicable for those alignments involving short query sequences only. Our method can be integrated into the CUDASW++ 3.0 package as an execution option and the length of the input query sequence is used to determine which method will be invoked for the alignments. If the length is not too long to fit all the required buffering into shared memory, our method is invoked. Otherwise, the original CUDASW++ 3.0 is invoked instead. Our method was originally designed based on CUDASW++ 2.0. To integrate our method into CUDASW++ 3.0, the PTX assembly instructions can be used in our proposed algorithm for further performance improvement.

### 4.4. Performance Evaluation of Different Threads and Blocks

**Figure 6:**
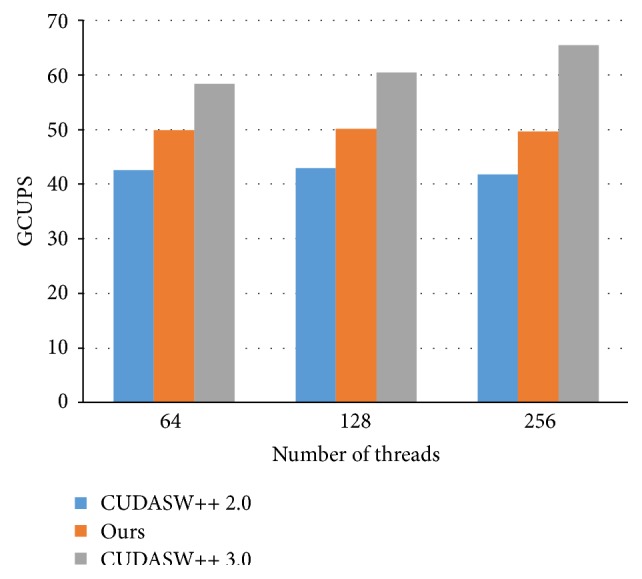
The performance analysis of 64 blocks based on different number of threads.

This subsection explores the influence of the numbers of threads and blocks. We take the query sequence, P86783, for the following study. First, we set the number of the blocks as 64 and change the number of threads, as shown in Figure 6. When the number of threads is increased, our approach and CUDASW++ 2.0 obtained almost the same GCUPS while CUDASW++ 3.0 has higher performance. When the number of threads per block becomes larger, the length deviation of the subject sequences per block becomes higher, resulting in poorer load balance between threads in the same block. Moreover, the amount of shared memory allocated to each thread is reduced when more threads in a block contend for the shared memory. On the other hand, more subject sequences per block can be aligned concurrently. For CUDASW++ 3.0, it adopts advanced scheduling designed especially for Kepler architecture, which prefers more threads per block.

**Figure 7:**
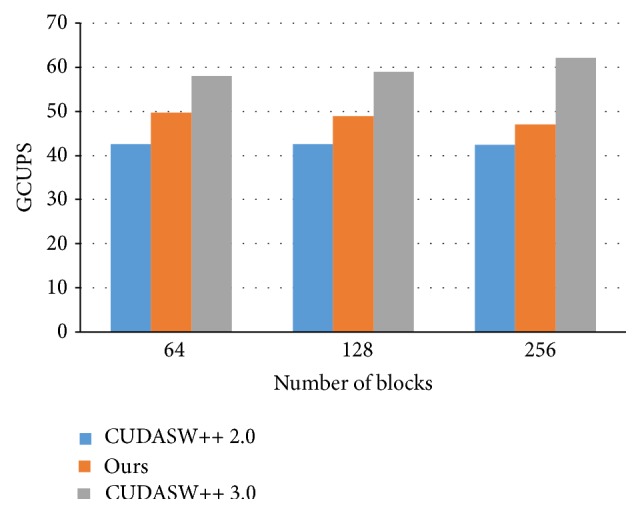
The performance analysis of 64 threads based on different number of blocks.

Next, we take the query sequence, P86783, to investigate the influence of the number of blocks, as shown in Figure 7. The number of threads per block is set to 64. When there are more blocks, it means that the total number of threads in a grid is increased. Consequently, the number of subject sequences allocated to each thread is decreased. On the other hand, we cannot run more than one block at the same time on any streaming multiprocessor since each block required almost all the shared memory space in its resident streaming multiprocessor. As a result, increasing the number of blocks incurs higher overhead for context switching between blocks.

## 5. Conclusions

The inexpensive general-purpose GPUs give engineers a great choice to accelerate time-consuming applications. In this work, we discussed how to use NVIDIA GPUs to implement the Smith-Waterman database search algorithm. We have added our method to the advanced package of CUDASW++ 2.0 as an option of execution. When users input a query sequence, the extended package will determine how to run the query based on the length of the query sequence as well as the space of shared memory per streaming multiprocessor. If the query sequence length is short with the calculation of the available shared memory per streaming multiprocessor, the extended package will use our method to run the query. Otherwise, the original CUDASW++ 2.0 will be used. Our idea can be applied to CUDASW++ 3.0 to improve the intertask kernel in the future.

We have evaluated our method on Tesla C1060 and K20 using the benchmark BLUSOM64. Further, we analyze the performance on different number of threads and blocks. The results suggested that the proposed method may improve Smith-Waterman algorithm on CUDA-enabled GPUs in proper allocation of block and thread numbers.

## Figures and Tables

**Pseudocode 1:**
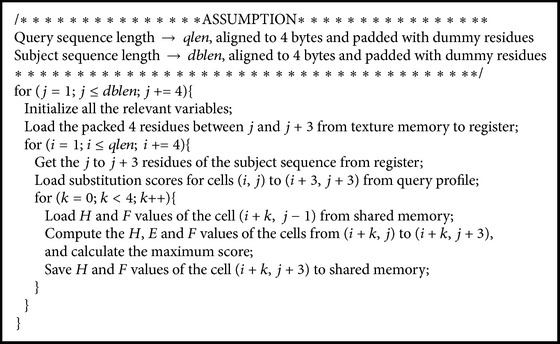
The pseudocode of the scoring function in our proposed method.

**Table 1:** The hardware configuration of the first experimental platform, where Tesla C1060 is included.

Intel Xeon processor E5504		NVIDIA Tesla C1060	
Number of CPUs	1	Number of GPUs	1
Number of processor cores	4	Number of processor cores	240
Clock speed	2 GHz	Clock speed	1.3 GHz
Memory size	6 GB	Memory size	4 GB
Memory types	DDR3 800	Memory types	GDDR3
Cache	4 MB	Memory clock	800 MHz

**Table 2:** The hardware configuration of the second experimental platform, where Tesla K20 is included.

Intel Core i7-4790		NVIDIA Tesla K20	
Number of CPUs	4	Number of GPUs	1
Number of processor cores	8	Number of processor cores	2496
Clock speed	3.6 GHz	Clock speed	0.71 GHz
Memory size	8 GB	Memory size	4.8 GB
Memory types	DDR3 1600	Memory types	GDDR5
Cache	8 MB	Memory clock	2600 MHz
